# Accurate Preoperative Diagnosis of Ovarian Pregnancy with Transvaginal Scan

**DOI:** 10.1155/2012/934571

**Published:** 2012-10-08

**Authors:** Natasha Gupta, Anu Gupta, Godwin Onyema, Yelena Pantofel, Shan-Ching Ying, Jack E. Garon, Charles Lampley, Josef Blankstein

**Affiliations:** ^1^Department of Obstetrics and Gynecology, Mount Sinai Hospital, 1500 S California Avenue, Chicago, IL 60608, USA; ^2^Department of Ultrasound, Mount Sinai Hospital, 1500 S California Avenue, Chicago, IL 60608, USA; ^3^Department of Pathology, Mount Sinai Hospital, 1500 S California Avenue, Chicago, IL 60608, USA

## Abstract

Ovarian pregnancy is a rare variant of ectopic pregnancy, and an accurate preoperative diagnosis is very challenging. Dr. Saint Monnissey described the first reported case of ovarian pregnancy in 17th century. Transvaginal ultrasonography is a valuable tool in identifying an ovarian pregnancy from other types of ectopic pregnancies. Management with laparoscopy or laparotomy is required in all cases, and in almost all cases, ovary can be preserved since implantation is usually superficial (Koo et al.; 2011). A single case of an ovarian pregnancy, consistent with Spiegelberg's criteria is presented here. This case history demonstrates the use of ultrasonography (USG) and surgery in the diagnosis and treatment, respectively, of the ovarian pregnancy. As we step into an era where in vitro fertilization rate is on its hike, one should be aware that incidence of ovarian pregnancy is also increasing, necessitating a high index of suspicion.

## 1. Introduction

Ovarian pregnancy still remains a diagnostic challenge. There are very few reports of an accurate preoperative diagnosis, utilizing sonography. The correct diagnosis is most frequently made at the surgery and requires histopathological confirmation. Diagnosis of ovarian pregnancy should be suspected from elevated beta hcg, lack of intrauterine gestation, a complex ovarian mass on USG, patient's risk factors, in addition to the Spiegelberg criteria. Patients most often undergo surgery for suspected tubal ectopic pregnancy or hemorrhagic corpus luteum. This case was correctly identified as ovarian gestation due to availability of high resolution ultrasonography, in absence of any significant symptoms or physical exam findings. This prevented potential rupture and thus a surgical emergency. 

## 2. Case Presentation

**Figure 1:**
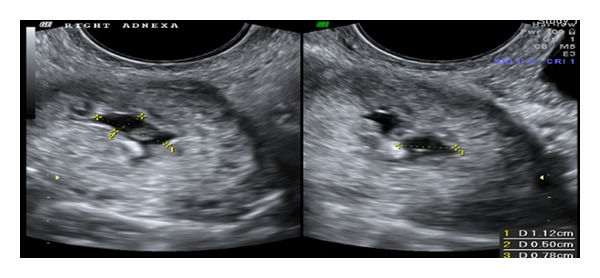


**Figure 2:**
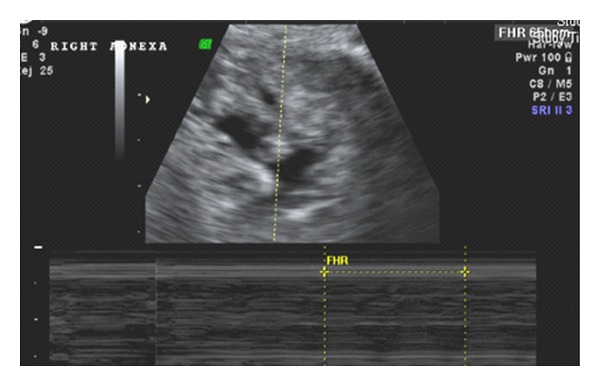


A 35-year-old primiparous, at 4+2/7 weeks gestation, estimated by her last menstrual period, presented to the emergency room, after her routine ultrasound as part of initial prenatal visit. The transvaginal ultrasound was suggestive of an empty anteverted uterus with homogenous echotexture, lack of intrauterine pregnancy, and an 8.6 mm endometrium stripe. It also showed a 4.3 × 3.1 × 2.8 cm complex mass (Figure 1) arising from the right ovary with a 1.1 × 0.7 × 0.5 cms gestational sac, yolk sac, and a 2.5 mm alive embryo with heart rate of 76 bpm within (Figure 2). The left ovary appeared unremarkable. Scant amount of free fluid was noted in the posterior cul de sac.

Patient denied any abdominal pain, vaginal bleeding, or any episode of syncope, dizziness, or shoulder pain. She declined any significant medical problems or medication use. She was a gravida three para zero, with history of one spontaneous abortion and one ectopic pregnancy, for which she underwent left salpingectomy three years back. Her surgical history was also significant for minilaparotomy for acute appendicitis, converted from laparoscopy. She was a smoker, with history of chlamydia treated four years back. Patient was also undergoing workup for secondary infertility for last two years, but denied the use of any ovulation induction agents. She received a hysterosalpingogram at infertility clinic, which showed a normal uterus, left fallopian tube stump and a severely diseased right fallopian tube, with complete occlusion and no peritoneal spillage.

Her abdomen was nontender, nondistended, and without any palpable masses. The pelvic examination revealed an anteverted, normal size, nontender uterus, and no adnexal tenderness or palpable adnexal masses. 

**Figure 3:**
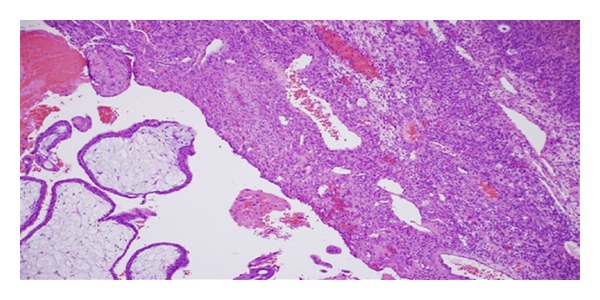
Histopathology revealed ovary with immature chorionic villi consistent with ectopic pregnancy. On the left lower field there are immature chorionic villi. On the right side is ovarian stroma with a primoridal follicle by the double arrows.

Vital signs were within normal limits. The quantitative beta hcg was 7744 mIU/mL. The hemoglobin level was at 12.7 gm/100 mL and the hematocrit was at 38.1%. A diagnosis of right ovarian gestation was made. Patient was informed of the diagnosis and a minilaparotomy was performed. Intraoperative findings revealed a normal right tube and fimbria, completely separate from the right ovary; a surgically absent left tube, a normal left ovary, minimal blood in the cul de sac, and a 1.5 cms nodular lesion on the right ovarian surface. A wedge resection of the nodular lesion was performed. The histopathological examination showed immature chorionic villi in the wall of the gestational sac (Figure 3), consistent with the ectopic ovarian gestation. The above mentioned intraoperative and the pathological findings fulfilled the Spiegelberg criteria [1], formulated in 1878, by Spiegelberg, for the correct diagnosis of ovarian pregnancy.

The immediate and long term postoperative recovery period was uneventful, and patient was discharged home after two days. 

## 3. Discussion

Primary ovarian pregnancy is a rare type of ectopic pregnancy, with an estimated incidence of 0.5–3% of all ectopic gestations. This incidence has increased substantially in recent years [2]. This increase may be attributable to increase in the detection rate, with the evolution of transvaginal sonography, a sensitive quantitative beta hcg, diagnostic laparoscopic surgeries for suspected ectopic gestation [3], and an increase in various risk factors associated with ovarian pregnancy.

Risk factors include endometriosis [4], sexually transmitted diseases, ovulation induction agents, tubal sterilization, intrauterine device use, and a history of abdominal surgery. Also, higher rates were noted for African American females and other minority groups; rates further increase progressively with age. In case of ovarian pregnancy, several case reports have been published indicating relationship between infertility treatments like in-vitro fertilization (IVF), embryo transfer, and intrauterine insemination (IUI). Common feature in all these procedures is enlargement of the ovary due to stimulation with gonadotropins [5]. Grimes et al. reported reproductive system pathology or infertility in more than 50% of his 24 cases [2]. The most common presenting symptoms include abdominal pain, vaginal bleeding, and amenorrhea, not unlike other ectopic gestations, although patient may present with the sole complaint of pelvic pain. An adnexal mass may be palpable on pelvic examination in 60% of cases. This patient was asymptomatic at presentation and was admitted from routine ultrasound. It is likely that patient had mild bleeding which she reported as her last period, thus making her gestational age more than just 4.2 weeks. She had a few risk factors like African American race, 35 years age, history of previous ectopic pregnancy on contralateral side, sexually transmitted disease, secondary infertility, and history of abdominal surgery.

Comstock et al. [6] reported a case series evaluating the ultrasonographic appearance of proven ovarian ectopic pregnancies. They showed a wide, echogenic ring with an internal echolucent area as compared to a thin tubal ring with tubal pregnancies or corpus luteum cyst; a yolk sac or fetal heart motion was also identified on occasion. It is rare to be able to correctly identify the embryo or trophoblastic tissue even intraoperatively. Benacerraf suggested that increasing the transducer frequency from 7 MHz to 10 MHz improves the diagnostic accuracy when an echolucent intrauterine collection of fluid is seen in early pregnancy [7]. Patel et al. reported a rare case of twin ovarian pregnancy diagnosed by ultrasound [8]. Other sonographic findings in various studies included complex adnexal masses [7, 8] or solid- cystic masses, with or without fluid in cul de sac, fluid surrounding the ovary, and ovarian enlargement. 

Ovarian pregnancies are very unlikely to be diagnosed preoperatively, as they may resemble any other ovarian cyst. Hallat [9] in his study of 25 cases of ovarian pregnancies reported that the most significant finding in his study was the inability to distinguish an ovarian pregnancy from a hemorrhagic ovary or ruptured corpus luteum. They are twice as likely to be diagnosed at surgery (mostly incidental, since patient is generally operated for a tubal ectopic or ruptured corpus luteal cyst) or following the pathological diagnosis [8]. Although ovarian pregnancies usually rupture by the 40th gestational day, there have been reports of these progressing into the third trimester and even to live births [10].

The most common surgical treatment consists of ovarian wedge resection or oophorectomy [8, 11], either laparoscopic or via minilaparotomy. The outcome of subsequent pregnancy is successful, with a low rate of subsequent ectopic pregnancy after the ovarian pregnancy is treated be surgery [4]. This patient was also taken for ovarian wedge resection via minilaparotomy versus laparoscopic due to patient's previous history of surgeries (especially previous conversion from laparoscopic to open) and pelvic inflammatory disease (PID). Although like other ectopic pregnancies, ovarian pregnancy can be treated with methotrexate [12], it is not known whether same criteria apply. Kudo et al. [12] first reported successful use of methotrexate to treat an ovarian pregnancy. Mittal et al. [13] successfully treated an ovarian pregnancy by injecting methotrexate into the sac at laparoscopy.

Most of the studies reported the use of laparoscopic treatment [3, 14]. In our patient, a presumptive diagnosis of ectopic pregnancy can be made based on the positive quantitative beta hcg, without an intrauterine gestation. However, given patient's lack of symptomatology, features of rupture, lack of palpable abdominal or pelvic masses and given the rarity of this condition, a preoperative diagnosis of ovarian pregnancy would be very difficult. In the absence of a very suggestive transvaginal scan, this patient may not have been taken for a surgery, thus leading to a potential surgical emergency like rupture.

High resolution transvaginal ultrasonography showing positive fetal heart rate, a gestational sac and yolk sac helped us to make an early and accurate diagnosis of an otherwise extremely rare entity. The intraoperative findings fulfilled all four of the Spiegelberg's criteria [1]: (1) an intact ipsilateral tube, clearly separate from the ovary; (2) a gestation occupying the normal position of the ovary; (3) a gestational sac connected to the uterus by the utero-ovarian ligament; (4) ovarian tissue in the wall of the gestational sac.
